# Supplementary material for: Aristolochic Acid I-Induced Hepatotoxicity in Tianfu Broilers Is Associated with Oxidative-Stress-Mediated Apoptosis and Mitochondrial Damage
Source: Animals (Basel). 2021 Dec 2;11(12):3437. doi: 10.3390/ani11123437 (PMC8698099; doi:10.3390/ani11123437)

Supplementary figure S1. The flow cytometry quadrant diagrams of ROS levels in liver.

A: CG group, B: LAG group, C: MAG group, D: HAG group.

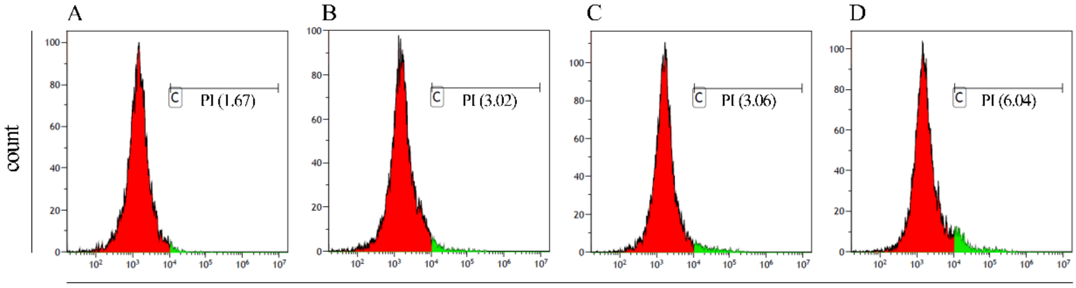

Supplementary figure S2. The flow cytometry quadrant diagrams of apoptosis in liver. A: CG group, B: LAG group, C: MAG group, D: HAG group.

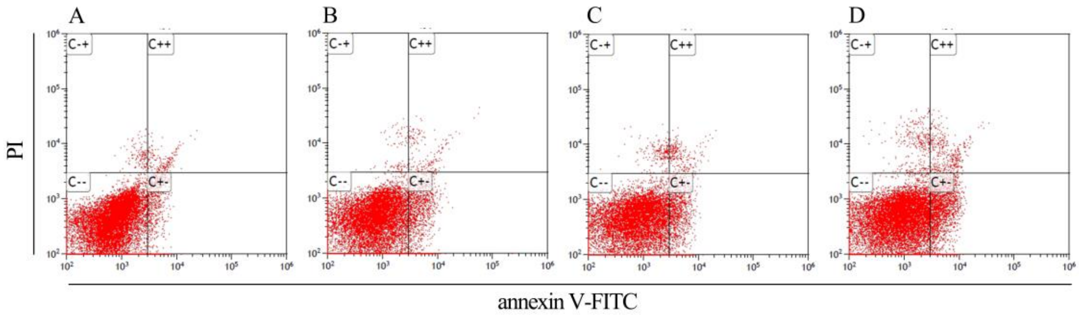

Supplementary figure S3. The flow cytometry quadrant diagrams of mitochondrial depolarization ratio in liver. A: CG group, B: LAG group, C: MAG group, D: HAG group.

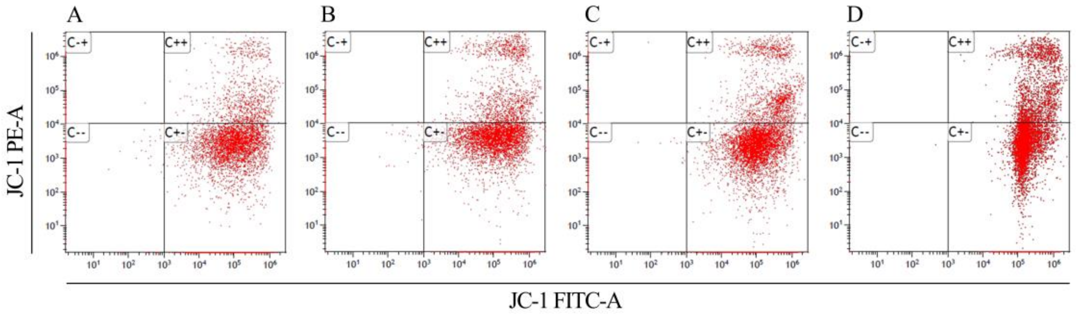

Supplement: Supplementary file 1 [file animals-11-03437-s001.zip › supplementary figures.pdf]
